# Stability of a Quartic Functional Equation

**DOI:** 10.1155/2014/752146

**Published:** 2014-01-23

**Authors:** Abasalt Bodaghi

**Affiliations:** Department of Mathematics, Garmsar Branch, Islamic Azad University, Garmsar, Iran

## Abstract

We obtain the general solution of the generalized quartic functional equation *f*(*x* + *my*) + *f*(*x* − *my*) = 2(7*m* − 9)(*m* − 1)*f*(*x*) + 2*m* ^2^(*m* ^2^ − 1)*f*(*y*)−(*m* − 1)^2^ *f*(2*x*) + *m* ^2^{*f*(*x* + *y*) + *f*(*x* − *y*)} for a fixed positive integer *m*. We prove the Hyers-Ulam stability for this quartic functional equation by the directed method and the fixed point method on real Banach spaces. We also investigate the Hyers-Ulam stability for the mentioned quartic functional equation in non-Archimedean spaces.

## 1. Introduction

We say a functional equation *ℱ* is stable if any function *f* satisfying the equation *ℱ* *approximately* is near to exact solution of *ℱ*. Moreover, a functional equation *ℱ* is hyperstable if any function *f* satisfying the equation *ℱ* *approximately* is a true solution of *ℱ*.

The study of stability problems for functional equations is related to a question of Ulam [1] concerning the stability of group homomorphisms, affirmatively answered for Banach spaces by Hyers [2]. Subsequently, the result of Hyers was generalized by a number of authors. For example, Bodaghi et al. investigated the Hyers-Ulam stability of Jordan ∗-derivation pairs for the Cauchy additive functional equation and the Cauchy additive functional inequality in [3]. For some results on the stability of various functional equations, see also [4–9].

The oldest quartic functional equation was introduced by Rassias in [10] and then was employed by other authors. Rassias [10] investigated stability properties of the following quartic functional equation:

(1) f(x+2y)+f(x−2y)+6f(x)  =4f(x+y)+4f(x−y)+24f(y).

Since *f*(2*x*) = 16*f*(*x*), we get

(2) f(x+2y)+f(x−2y)=10f(x)+24f(y)−f(2x)+4f(x+y)+4f(x−y).

In [11], Chung and Sahoo determined the general solution of (2) without assuming any regularity conditions on the unknown function. Indeed, they proved that the function *f* : ℝ → ℝ is a solution of (2) if and only if *f*(*x*) = *Q*(*x*, *x*, *x*, *x*) where the function *Q* : ℝ^4^ → ℝ is symmetric and additive in each variable. The fact that every solution of (2) is even implies that it can be written as follows:

(3) f(2x+y)+f(2x−y)=24f(x)−6f(y)+4f(x+y)+4f(x−y).

Lee et al. [12] obtained the general solution of (3) and proved the Hyers-Ulam stability of this equation. Also Park [13] investigated the stability problem of (3) in the orthogonality normed space. Lee and Chung [14] considered the following quartic functional equation, which is a generalization of (3):

(4) f(mx+y)+f(mx−y) =2m2(m2−1)f(x)−2(m2−1)f(y)  +m2f(x+y)+m2f(x−y)

for fixed integer *m* with *m* ≠ 0, ±1. They obtained the general solution of (4) and proved its Hyers-Ulam stability.

Bodaghi et al. [15] applied the fixed point alternative theorem (Theorem 8 of the current paper) to establish Hyers-Ulam stability of (3). They also showed that the functional equation (3) can be hyperstable under some conditions. This method which is different from the “*direct method,*” initiated by Hyers in 1941, had been applied by Cădariu and Radu for the first time in [16]. In other words, they employed this fixed point method to the investigation of the Cauchy functional equation [17] and of the quadratic functional equation [16] (for more applications of this method, see [18–20]).

In this paper, we consider the following functional equation which is somewhat different from (2), (3), and (4):

(5) f(x+my)+f(x−my)  =2(7m−9)(m−1)f(x)+2m2(m2−1)f(y)   −(m−1)2f(2x)+m2{f(x+y)+f(x−y)}

for a fixed positive integer *m*. In case *m* = 2, then (5) is the celebrated Jordan-von Neumann equation. Then we find out the general solution of (5). We also prove the Hyers-Ulam stability problem and the hyperstability for (5) by the directed method and the fixed point method.

## 2. General Solution of (5)

To achieve our aim in this section, we need the following lemma.

**Lemma 1**

Let *𝒳* and *𝒴* be real vector spaces. If a function *f* : *𝒳* → *𝒴* satisfies the functional equation (5) for all integers *m* ≥ 3, then *f* satisfies *f*(*mx*) = *m* ^4^ *f*(*x*) for all integers *m* ≥ 2.

**Proof**

Letting *x* = *y* = 0 in (5), we get *f*(0) = 0. Once more, by putting *y* = 0 in (5), we obtain

(6) $$\begin{array}{c} f\left(2x\right)=2^{4}f\left(x\right). \end{array}$$

In the case that *m* = 3, by replacing *x*,  *y* by 3*x*,  *x* in (5), respectively, we have

(7) f(6x)=48f(3x)+144f(x)−4f(6x)+9{f(4x)+f(2x)}.

The above equality and (6) imply that *f*(3*x*) = 3^4^ *f*(*x*). Now, assume that, for every *k* ≤ *m* − 1, we have *f*(*kx*) = *k* ^4^ *f*(*x*). If *m* = 2*n*, then *f*(*mx*) = *f*(2*nx*) = 2^4^ *f*(*nx*). Since *n* ≤ *m* − 1, we have *f*(*nx*) = *n* ^4^ *f*(*x*), and thus *f*(*mx*) = *m* ^4^ *f*(*x*). Let *m* = 2*n* + 1. Then, by substituting *x*,  *y* by *mx*,  *x* in (5), respectively, we have

(8) f(2mx)=2(7m−9)(m−1)f(mx)+2m2(m2−1)f(x)−(m−1)2f(2mx)+m2{f((m+1)x)+f((m−1)x)}.

Since *m* = 2*n* + 1, we have *m* + 1 = 2(*n* + 1) and *m* = 2*n*. Replacing these equalities in (8) and using (6), we get *f*(*mx*) = *m* ^4^ *f*(*x*). This completes the proof.

**Remark 2**

It is shown in [21, Lemma 2.1] that a mapping *f* : *𝒳* → *𝒴* satisfies the functional equation (1) if and only if *f* satisfies

(9) f(x+my)+f(x−my) =2m2(m2−1)f(y)−2(m2−1)f(x)  +m2{f(x+y)+f(x−y)}.

There is a gap in its proof. In fact, in the proof, the author only showed that the functional equation (1) implies (9) but the converse is not proved.  Theorem 3 resolved this problem. Indeed, we solve the equation of (5).

**Theorem 3**

Let *𝒳* and *𝒴* be real vector spaces. Then a mapping *f* : *𝒳* → *𝒴* satisfies the functional equation (2) if and only if it satisfies (5) where *m* ≥ 3. Therefore, every solution of the functional equation (5) is also a quartic mapping.

**Proof**

Suppose that *f* : *𝒳* → *𝒴* satisfies the functional equation (2). Putting *x* = *y* = 0 in (2), we get *f*(0) = 0. Let *y* = 0 in (2) to get *f*(2*x*) = 16*f*(*x*) for all *x* ∈ *𝒳*. Setting *x* = 0 in (2) and using the fact that *f*(*y*) = 16*f*(*y*), we have *f*(−*y*) = *f*(*y*). Letting *y* = *x* in (2), we have *f*(3*x*) = 81*f*(*x*) for all *x* ∈ *𝒳*. By induction, we obtain *f*(*kx*) = *k* ^4^ *f*(*x*) for all positive integers *k*. Replacing *x* by *x* + *y* and *x* − *y* in (2), respectively, we have

(10) f(x+3y)+f(x−3y)=48f(x)+144f(y)−4f(2x)+9{f(x+y)+f(x−y)}.

In Similar way to the above, we get

(11) f(x+4y)+f(x−4y)=114f(x)+480f(y)−9f(2x)+16{f(x+y)+f(x−y)}.

Using the above method, we can deduce that

(12) f(x+my)+f(x−my) =amf(x)+bmf(y)−(m−1)2f(2x)  +m2{f(x+y)+f(x−y)}

for which

(13) $$\begin{array}{c} a_{m}=-a_{m-2}+28m^{2}-120m+156,\\ a_{2}=10,\;\;a_{3}=48,\\ b_{m}=2b_{m-1}-b_{m-2}+24{\left(m-1\right)}^{2},\\ b_{2}=24,\;\;b_{3}=144. \end{array}$$

Solving the above recurrence equations is routine, and so we get

(14) $$\begin{array}{c} a_{m}=2\left(7m-9\right)\left(m-1\right),\;\;b_{m}=2m^{2}\left(m^{2}-1\right) \end{array}$$

for all *x*, *y* ∈ *𝒳* and each positive integer *m* ≥ 2.

Conversely, assume that *f* : *𝒳* → *𝒴* satisfies the functional equation for each *k* ≥ *m*, in particular, for *k* = *m*(*m* − 1). Hence for each *x*, *y* ∈ *𝒳*, we have

(15) f(x+m(m−1)y)+f(x−m(m−1)y) =2(7m−9)(m−1)f(x)+2m2(m2−1)f((m−1)y)  −(m−1)2f(2x)+m2{f(x+(m−1)y)               +f(x−(m−1)y)}.

By Lemma 1, we have *f*((*m* − 1)*y*) = (*m* − 1)^4^ *f*(*y*) and so

(16) f(x+m(m−1)y)+f(x−m(m−1)y) =2(7m−9)(m−1)f(x)+2m2(m2−1)(m−1)4f(y)  −(m−1)2f(2x)+m2{f(x+(m−1)y)               +f(x−(m−1)y)}.

On the other hand,

(17) f(x+(m2−m)y)+f(x−(m2−m)y) =2(7(m2−m)−9)((m2−m)−1)f(x)  +2(m2−m)2((m2−m)2−1)f(y)  −((m2−m)−1)2f(2x)  +(m2−m)2{f(x+y)+f(x−y)}.

Using (16) and (17), we get

(18) m2{f(x+(m−1)y)+f(x−(m−1)y)} =2[(7(m2−m)−9)((m2−m)−1)    −(7m−9)(m−1)]f(x)  +2[(m2−m)2((m2−m)2−1)     −m2(m2−1)(m−1)4]f(y)  −[((m2−m)−1)2−(m−1)2]f(2x)  +m2(m−1)2{f(x+y)+f(x−y)}.

A calculation shows that

(19) f(x+(m−1)y)+f(x−(m−1)y) =2(7m−16)(m−2)f(x)  +2(m−1)2((m−1)2−1)f(y)−(m−2)2f(2x)  +(m−1)2{f(x+y)+f(x−y)}.

Thus, if *f* satisfies the functional equation (5) for all *m* ≥ 3, then it satisfies (5) for *m* − 1. In particular, *f* satisfies (2).

## 3. Hyers-Ulam Stability of (5)

Let *m* be an integer with *m* ≥ 2. We use the abbreviation for the given mapping *f* : *𝒳* → *𝒴* as follows:

(20) 𝒟mf(x,y) :=f(x+my)+f(x−my)   −2(7m−9)(m−1)f(x)−2m2(m2−1)f(y)   +(m−1)2f(2x)−m2{f(x+y)+f(x−y)}                      (x,y∈𝒳).

Throughout this section, we assume that *𝒳* is a normed real linear space with norm ||·||_*𝒳*_ and *𝒴* is a real Banach space with norm ||·||_*𝒴*_. We are going to prove the stability of the quartic functional equation (5).

**Theorem 4**

Let *α* be a real number and let *f* : *𝒳* → *𝒴* be a mapping for which there exists a function *ϕ* : *𝒳* × *𝒳* → [−*α*, *∞*) such that

(21) $$\begin{array}{c} \tilde{\phi }\left(x,y\right):=\sum_{k=0}^{\infty }\frac{1}{2^{4k}}\phi \left(2^{k}x,2^{k}y\right)<\infty , \end{array}$$

(22) $$\begin{array}{c} {||{𝒟}_{m}f\left(x,y\right)||}_{𝒴}\leq \alpha +\phi \left(x,y\right) \end{array}$$

for all *x*, *y* ∈ *𝒳*, where *m* is an integer with *m* ≥ 2. Then there exists a unique quartic mapping *𝒯* : *𝒳* → *𝒴* such that

(23) ||f(x)−𝒯(x)−2m2(m+1)15(m−1)f(0)||𝒴 ≤α15(m−1)2+ϕ~(x,0)16(m−1)2

for all *x* ∈ *𝒳*.

**Proof**

Putting *y* = 0 in (22), we have

(24) ||(m−1)2f(2x)−16(m−1)2f(x)−2m2(m2−1)f(0)||𝒴 ≤α+ϕ(x,0)

for all *x* ∈ *𝒳*. Thus

(25) ||116f(2x)−f(x)−m2(m+1)8(m−1)f(0)||𝒴 ≤α16(m−1)2+ϕ(x,0)16(m−1)2

for all *x* ∈ *𝒳*. Replacing *x* by 2*x* in (25) and continuing this method, we get

(26) ||f(2nx)24n−f(x)−m2(m+1)8(m−1)∑k=0n−1124kf(0)||𝒴 ≤116∑k=0n−1α24k(m−1)2+116∑k=0n−1ϕ(2kx,0)24k(m−1)2.

On the other hand, we can use induction to obtain

(27) ||f(2nx)24n−f(2lx)24l||𝒴≤m2(m+1)8(m−1)∑k=ln−1124k||f(0)||𝒴+116∑k=ln−1α24k(m−1)2+116∑k=ln−1ϕ(2kx,0)24k(m−1)2

for all *x* ∈ *𝒳*, and *n* > *l* ≥ 0. Thus the sequence {*f*(2^*n*^ *x*)/2^4*n*^} is Cauchy by (21) and (27). Completeness of *𝒴* allows us to assume that there exists a map *𝒯* so that

(28) $$\begin{array}{c} \underset{n\to \infty }{\lim}\frac{f\left(2^{n}x\right)}{2^{4n}}=𝒯\left(x\right). \end{array}$$

Taking the limit as *n* → *∞* in (26) and applying (28), we can see that inequality (23) holds. Now, we replace *x*,  *y* by 2^*n*^ *x*,  2^*n*^ *y*, respectively, in (22); then

(29) $$\begin{array}{c} \frac{1}{2^{4n}}{||{𝒟}_{m}f\left(2^{n}x,2^{n}y\right)||}_{𝒴}\leq \frac{1}{2^{4n}}\alpha +\frac{\phi \left(2^{n}x,2^{n}y\right)}{2^{4n}}. \end{array}$$

Letting the limit as *n* → *∞*, we obtain *𝒟*_*m*_ *𝒯*(*x*, *y*) = 0 for all positive integers *m* ≥ 2 and all *x*, *y* ∈ *𝒳*. Hence, by Theorem 3, it indicates that *𝒯* : *𝒳* → *𝒴* is a quartic mapping. Now, let *𝒯*′ : *𝒳* → *𝒴* be another quartic mapping satisfying (23). Then we have

(30) ||𝒯(x)−𝒯′(x)||𝒴 =124n||𝒯(2nx)−𝒯′(2nx)||𝒴 ≤124n(||𝒯(2nx)−f(2nx)+2m2(m+1)15(m−1)f(0)||𝒴     +||f(2nx)−𝒯′(2nx)−2m2(m+1)15(m−1)f(0)||𝒴) ≤124n[α15(m−1)2+ϕ~(x)16(m−1)2] =α15(m−1)224n+116∑k=0∞124(n+k)ϕ(2n+kx,0) =α15(m−1)224n+116∑k=0∞124nϕ(2nx,0)

for all *x* ∈ *𝒳*. Taking *n* → *∞* in the preceding inequality, we immediately find the uniqueness of *𝒯*. This completes the proof.

**Corollary 5**

Let *α*, *β*, *γ*, *r*, and *s* be nonnegative real numbers such that *s* > 0 and *r*, *s* < 4. Suppose that *f* : *𝒳* → *𝒴* is a mapping fulfilling

(31) $$\begin{array}{c} {||{𝒟}_{m}f\left(x,y\right)||}_{𝒴}\leq \alpha +\beta {||x||}_{𝒳}^{r}+\gamma {||y||}_{𝒳}^{s} \end{array}$$

for all *x*, *y* ∈ *𝒳*, where *m* is an integer with *m* ≥ 2. Then there exists a unique quartic mapping *𝒯* : *𝒳* → *𝒴* such that

(32) ||f(x)−𝒯(x)||𝒴≤2α15(m−1)2−α2m4+13m2−30m+15+β(m−1)2(24−2r)||x||𝒳r

for all  *x* ∈ *𝒳*  and all *x* ∈ *𝒳*∖{0} if *r* < 0.

**Proof**

Setting *ϕ*(*x*, *y*) = *β*||*x*||_*𝒳*_^*r*^ + *γ*||*y*||_*𝒳*_^*s*^ in Theorem 12, we have

(33) ||f(x)−𝒯(x)−2m2(m+1)15(m−1)f(0)||𝒴 ≤α15(m−1)2+β(m−1)2(24−2r)||x||𝒳r.

It follows from (31) that

(34) $$\begin{array}{c} {||f\left(0\right)||}_{𝒴}\leq \frac{\alpha }{2m^{4}+13m^{2}-30m+15}. \end{array}$$

By these statements we can get the result.

We have the following result which is analogous to Theorem 12 for the quartic functional equation (5). We include its proof.

**Theorem 6**

Suppose that *f* : *𝒳* → *𝒴* is a mapping for which there exists a function *ϕ* : *𝒳* × *𝒳* → [0, *∞*) such that

(35) $$\begin{array}{c} \tilde{\phi }\left(x,y\right):=\sum_{k=1}^{\infty }2^{4k}\phi \left(\frac{x}{2^{k}},\frac{y}{2^{k}}\right)<\infty , \end{array}$$

(36) $$\begin{array}{c} {||{𝒟}_{m}f\left(x,y\right)||}_{𝒴}\leq \phi \left(x,y\right) \end{array}$$

for all *x*, *y* ∈ *𝒳*, where *m* is an integer with *m* ≥ 2. Then there exists a unique quartic mapping *𝒯* : *𝒳* → *𝒴* such that

(37) $$\begin{array}{c} {||f\left(x\right)-𝒯\left(x\right)||}_{𝒴}\leq \frac{\tilde{\phi }\left(x,0\right)}{{\left(m-1\right)}^{2}} \end{array}$$

for all *x* ∈ *𝒳*.

**Proof**

It follows from (35) that *ϕ*(0,0) = 0. Thus from (36) we have *f*(0) = 0. Putting *y* = 0 in (36), we get

(38) $$\begin{array}{c} {||{\left(m-1\right)}^{2}f\left(2x\right)-16{\left(m-1\right)}^{2}f\left(x\right)||}_{𝒴}\leq \phi \left(x,0\right) \end{array}$$

for all *x* ∈ *𝒳*. If we replace *x* by *x*/2 in the above inequality and divide both sides by (*m* − 1)^2^, we have

(39) $$\begin{array}{c} {||f\left(x\right)-16f\left(\frac{x}{2}\right)||}_{𝒴}\leq \frac{\phi \left(x/2,0\right)}{{\left(m-1\right)}^{2}}. \end{array}$$

Using triangular inequality and proceeding this way, we obtain

(40) $$\begin{array}{c} {||f\left(x\right)-2^{4n}f\left(\frac{x}{2^{n}}\right)||}_{𝒴}\leq \frac{1}{{\left(m-1\right)}^{2}}\sum_{k=1}^{n}2^{4k}\phi \left(\frac{x}{2^{k}},0\right) \end{array}$$

for all *x* ∈ *𝒳*. If we show that the sequence {2^4*n*^ *f*(*x*/2^*n*^)} is Cauchy, then it will be convergent by the completeness of *𝒴*. For this, if we replace *x* by *x*/2^*l*^ in (40) and then multiply both sides by 2^4*l*^, then we get

(41) ||24(l+n)f(x2n)−24lf(x2l)||𝒴 ≤1(m−1)2∑k=1n24(k+l)ϕ(x2k+l,0) =1(m−1)2∑k=l+1l+n24kϕ(x2k,0)

for all *x* ∈ *𝒳*, and *n* > *l* > 0. Thus the mentioned sequence is convergent to the mapping *𝒯*; that is,

(42) $$\begin{array}{c} 𝒯\left(x\right):=\underset{n\to \infty }{\lim}2^{4n}f\left(\frac{x}{2^{n}}\right). \end{array}$$

Now, in a similar way to the proof of Theorem 12, we can complete the rest of the proof.

**Corollary 7**

Let *β*, *γ*, *r*, and *s* be nonnegative real numbers such that *r*, *s* > 4. Suppose that *f* : *𝒳* → *𝒴* is a mapping fulfilling

(43) $$\begin{array}{c} {||{𝒟}_{m}f\left(x,y\right)||}_{𝒴}\leq \beta {||x||}_{𝒳}^{r}+\gamma {||y||}_{𝒳}^{s} \end{array}$$

for all *x*, *y* ∈ *𝒳*, where *m* is an integer with *m* ≥ 2. Then there exists a unique quartic mapping *𝒯* : *𝒳* → *𝒴* such that

(44) $$\begin{array}{c} {||f\left(x\right)-𝒯\left(x\right)||}_{𝒴}\leq \frac{\beta }{{\left(m-1\right)}^{2}\left(2^{r}-2^{4}\right)}{||x||}_{𝒳}^{r} \end{array}$$

for all *x* ∈ *𝒳*.

**Proof**

First, we note that if we put *x* = *y* = 0 in (43), we have *f*(0) = 0. Taking *ϕ*(*x*, *y*) = *β*||*x*||_*𝒳*_^*r*^ + *γ*||*y*||_*𝒳*_^*s*^ in Theorem 14, we can obtain the desired result.

We are going to investigate the hyperstability of the given quartic functional equation (5) by using the fixed point method. First, we bring the next theorem which was proved in [22]. This result plays a fundamental role to achieve our goal.

**Theorem 8 (the fixed point alternative theorem)**

Let (Δ, *d*) be a complete generalized metric space and let *𝒥* : Δ → Δ be a mapping with Lipschitz constant *L* < 1. Then, for each element *α* ∈ Δ, either *d*(*𝒥* ^*n*^ *α*, *𝒥* ^*n*+1^ *α*) = *∞* for all *n* ≥ 0 or there exists a natural number *n*_0_ such that

- *d*(*𝒥* ^*n*^ *α*, *𝒥* ^*n*+1^ *α*) < *∞* for all *n* ≥ *n*_0_;
- the sequence {*𝒥* ^*n*^ *α*} is convergent to a fixed point β* of *𝒥*;
- β* is the unique fixed point of *𝒥* in the set Δ_1_ = {*β* ∈ Δ : *d*(*𝒯* ^*n*_0_^ *α*, *β*) < *∞*};
- *d*(*β*, β*)≤(1/(1 − *L*))*d*(*β*, *𝒥β*) for all *β* ∈ Δ_1_.

**Theorem 9**

Let *f* : *𝒳* → *𝒴* be a mapping with *f*(0) = 0 and let *φ* : *𝒳* × *𝒳* → [0, *∞*) be a function such that

(45) $$\begin{array}{c} {||{𝒟}_{m}f\left(x,y\right)||}_{𝒴}\leq \varphi \left(x,y\right) \end{array}$$

for all *x*, *y* ∈ *𝒳*, where *m* is an integer with *m* ≥ 2. If there exists a constant *M* ∈ (0,1), such that

(46) $$\begin{array}{c} \varphi \left(2x,2y\right)\leq 16M\varphi \left(x,y\right) \end{array}$$

for all *x*, *y* ∈ *𝒳*, then there exists a unique quartic mapping *𝒯* : *𝒳* → *𝒴* such that

(47) $$\begin{array}{c} {||f\left(x\right)-𝒯\left(x\right)||}_{𝒴}\leq \frac{1}{16{\left(m-1\right)}^{2}\left(1-M\right)}\varphi \left(x,0\right) \end{array}$$

for all *x* ∈ *𝒳*.

**Proof**

We wish to make the conditions of Theorem 8. We consider the set

(48) $$\begin{array}{c} \Delta =\left\{g:𝒳⟶𝒴\;|\;g\left(0\right)=0\right\} \end{array}$$

and define the mapping *𝔇* on Δ × Δ as follows:

(49) 𝔇(g,h):=inf⁡{C∈(0,∞):||g(x)−h(x)||𝒴          ≤Cφ(x,0),  (∀x∈𝒳)},

if there exists such constant *C*, and *𝔇*(*g*, *h*) = *∞*, otherwise. In a similar way to the proof of [15, Theorem 2.2], we can show that *𝔇* is a generalized metric on Δ and the metric space (Δ, *𝔇*) is complete. Here, we define the mapping *𝒥* : Δ → Δ by

(50) $$\begin{array}{c} 𝒥h\left(x\right)=\frac{1}{16}h\left(2x\right),\;\left(x\in 𝒳\right). \end{array}$$

If *g*, *h* ∈ Δ such that *𝔇*(*g*, *h*) < *C*, by definitions of *𝔇* and *𝒥*, we have

(51) $$\begin{array}{c} {||\frac{1}{16}g\left(2x\right)-\frac{1}{16}h\left(2x\right)||}_{𝒴}\leq \frac{1}{16}C\varphi \left(2x,0\right) \end{array}$$

for all *x* ∈ *𝒳*. Using (46), we get

(52) $$\begin{array}{c} {||\frac{1}{16}g\left(2x\right)-\frac{1}{16}h\left(2x\right)||}_{𝒴}\leq CM\varphi \left(x,0\right) \end{array}$$

for all *x* ∈ *𝒳*. The above inequality shows that *𝔇*(*𝒥g*, *𝒥h*) ≤ *M𝔇*(*g*, *h*) for all *g*, *h* ∈ Δ. Hence, *𝒥* is a strictly contractive mapping on Δ with a Lipschitz constant *M*. We now show that *𝔇*(*𝒥f*, *f*) < *∞*. Putting *y* = 0 in (45), we obtain

(53) $$\begin{array}{c} {||\frac{1}{16}f\left(2x\right)-f\left(x\right)||}_{𝒴}\leq \frac{\varphi \left(x,0\right)}{16{\left(m-1\right)}^{2}} \end{array}$$

for all *x* ∈ *𝒳*. We conclude from the last inequality that

(54) $$\begin{array}{c} 𝔇\left(𝒥f,f\right)\leq \frac{1}{16{\left(m-1\right)}^{2}}. \end{array}$$

Theorem 8 shows that *𝔇*(*𝒥* ^*n*^ *g*, *𝒥* ^*n*+1^ *g*) < *∞* for all *n* ≥ 0, and thus in this theorem we have *n*_0_ = 0. Consequently, the parts (iii) and (iv) of Theorem 8 hold on the whole Δ. Hence there exists a unique mapping *𝒯* : *𝒳* → *𝒴* such that *𝒯* is a fixed point of *𝒥* and that *𝒥* ^*n*^ *f* → *𝒯* as *n* → *∞*. Thus

(55) $$\begin{array}{c} \underset{n\to \infty }{\lim}\frac{f\left(2^{n}x\right)}{2^{4n}}=𝒯\left(x\right) \end{array}$$

for all *x* ∈ *𝒳*, and so

(56) $$\begin{array}{c} d\left(f,𝒯\right)\leq \frac{1}{1-M}d\left(𝒥f,f\right)\leq \frac{1}{16{\left(m-1\right)}^{2}\left(1-M\right)}. \end{array}$$

The above inequalities show that (47) is true for all *x* ∈ *𝒳*. Now, it follows from (46) that

(57) $$\begin{array}{c} \underset{n\to \infty }{\lim}\frac{\varphi \left(2^{n}x,2^{n}x\right)}{2^{4n}}=0. \end{array}$$

Substituting *x* and *y* by 2^*n*^ *x* and 2^*n*^ *y*, respectively, in (45), we get

(58) $$\begin{array}{c} \frac{1}{2^{4n}}{||{𝒟}_{m}f\left(2^{n}x,2^{n}y\right)||}_{𝒴}\leq \frac{\varphi \left(2^{n}x,2^{n}y\right)}{2^{4n}}. \end{array}$$

Taking the limit as *n* → *∞*, we obtain *𝒟*_*m*_ *𝒯*(*x*, *y*) = 0 for all integers  *m* ≥ 2  and all  *x*, *y* ∈ *𝒳*. It follows from Theorem 3 that *𝒯* : *𝒳* → *𝒴* is a quartic mapping which is unique.

**Corollary 10**

Let *α*, *β*, and *r* be nonnegative real numbers with *r*, *s* < 4 and let *f* : *𝒳* → *𝒴* be a mapping such that

(59) $$\begin{array}{c} {||{𝒟}_{m}f\left(x,y\right)||}_{𝒴}\leq \alpha {||x||}_{𝒳}^{r}+\beta {||y||}_{𝒳}^{r} \end{array}$$

for all *x*, *y* ∈ *𝒳*. Then there exists a unique quartic mapping *𝒯* : *𝒳* → *𝒴* satisfying

(60) $$\begin{array}{c} {||f\left(x\right)-𝒯\left(x\right)||}_{𝒴}\leq \frac{\alpha }{16{\left(m-1\right)}^{2}\left(2^{4}-2^{r}\right)}{||x||}_{𝒳}^{r} \end{array}$$

for all *x* ∈ *𝒳*.

**Proof**

Note that inequality (59) implies that *f*(0) = 0. If we put *φ*(*x*, *y*) = *α*||*x*||_*𝒳*_^*r*^ + *β*||*y*||_*𝒳*_^*r*^ in Theorem 9, we obtain the desired result.

In the next result, we prove the hyperstability of quartic functional equations under some conditions.

**Corollary 11**

Let *r*, *s*, and *α* be nonnegative real numbers with 0 < *r* + *s* ≠ 4 and let *f* : *𝒳* → *𝒴* be a mapping such that

(61) $$\begin{array}{c} {||{𝒟}_{m}f\left(x,y\right)||}_{𝒴}\leq \alpha {||x||}_{𝒳}^{r}{||y||}_{𝒳}^{s} \end{array}$$

for all *x*, *y* ∈ *𝒳*. Then *f* is a quartic mapping on *𝒳*.

**Proof**

Putting *x* = *y* = 0 in (61), we get *f*(0) = 0. Again, if we put *y* = 0 in (61), then we have *f*(2*x*) = 16*f*(*x*) for all *x* ∈ *𝒳*. It is easy to check that *f*(2^*n*^ *x*) = 2^4*n*^ *f*(*x*), and so *f*(*x*) = *f*(2^*n*^ *x*)/2^4*n*^ for all *x* ∈ *𝒳* and *n* ∈ *ℕ*. Now, it follows from Theorem 9 that *f* is a quartic mapping when *φ*(*x*, *y*) = *α*||*x*||_*𝒳*_^*r*^||*y*||_*𝒳*_^*s*^.

## 4. Stability of (5) in Non-Archimedean Spaces

We recall some basic facts concerning non-Archimedean spaces and some preliminary results. By a non-Archimedean field we mean a field *𝕂* equipped with a function (valuation) |·| from *𝕂* into [0, *∞*) such that |*r*| = 0 if and only if *r* = 0, |*rs*| = |*r*||*s*|, and |*r* + *s*|≤ max{|*r*|, |*s*|} for all *r*, *s* ∈ *𝕂*. Clearly |1| = |−1| = 1 and |*n*| ≤ 1 for all *n* ∈ *ℕ*.

Let *𝒳* be a vector space over a scalar field *𝕂* with a non-Archimedean nontrivial valuation |·|. A function ||·|| : *𝒳* → ℝ is a non-Archimedean norm (valuation) if it satisfies the following conditions:

(i) ||*x*|| = 0 if and only if *x* = 0;
(ii) ||*rx*|| = |*r*|||*x*||, (*x* ∈ *𝒳*, *r* ∈ *𝕂*);
(iii) the strong triangle inequality (ultrametric); namely,
  (62) $$\begin{array}{c} ||x+y||\leq \max\left\{||x||,||y||\right\},\;\left(x,y\in 𝒳\right). \end{array}$$

Then (*𝒳*, ||·||) is called a non-Archimedean space. Due to the fact that

(63) $$\begin{array}{c} ||x_{n}-x_{m}||\leq \max\left\{||x_{j+1}-x_{j}||;m\leq j\leq n-1\right\},\;\left(n\geq m\right) \end{array}$$

a sequence {*x*_*n*_} is Cauchy if and only if {*x*_*n*+1_ − *x*_*n*_} converges to zero in a non-Archimedean normed space *𝒳*. By a complete non-Archimedean normed space we mean one in which every Cauchy sequence is convergent.

In [23], Hensel discovered the *p*-adic numbers as a number theoretical analogue of power series in complex analysis. The most interesting example of non-Archimedean spaces is *p*-adic numbers. A key property of *p*-adic numbers is that they do not satisfy the Archimedean axiom: for all *x*, *y* > 0, there exists an integer *n* such that *x* < *ny*.

Let *p* be a prime number. For any nonzero rational number *x* = *p* ^*r*^(*m*/*n*) in which *m* and *n* are coprime to the prime number *p*. Consider the *p*-adic absolute value |*x*|_*p*_ = *p* ^*r*^ on *ℚ*. It is easy to check that |·| is a non-Archimedean norm on *ℚ*. The completion of *ℚ* with respect to |·| which is denoted by *ℚ*_*p*_ is said to be the *p*-adic number field. One should remember that if *p* > 3, then |2^*n*^| = 1 for all integers *n*. In [24], the stability of some functional equations in non-Archimedean normed spaces is investigated (see also [25]).

Here and subsequently, we assume that *𝒳* is a normed space and *𝒴* is a complete non-Archimedean space unless otherwise stated explicitly. In the upcoming theorem, we prove the stability of the functional equation (5).

**Theorem 12**

Let *ϕ* : *𝒳* × *𝒳* → [0, *∞*) such that

(64) $$\begin{array}{c} \underset{k\to \infty }{\lim}\frac{1}{{\left|16\right|}^{k}}\phi \left(2^{k}x,2^{k}y\right)=0 \end{array}$$

for all *x*, *y* ∈ *𝒳*. Suppose that *f* : *𝒳* → *𝒴* is a mapping satisfying the equality

(65) $$\begin{array}{c} ||{𝒟}_{m}f\left(x,y\right)||\leq \phi \left(x,y\right) \end{array}$$

for all *x*, *y* ∈ *𝒳*, where *m* is an integer with *m* ≥ 2. Then there exists a unique quartic mapping *Q* : *𝒳* → *𝒴* such that

(66) $$\begin{array}{c} ||f\left(x\right)-Q\left(x\right)||\leq \frac{1}{\left|16{\left(m-1\right)}^{2}\right|}\tilde{\phi }\left(x\right) \end{array}$$

for all *x* ∈ *𝒳* where $\tilde{\phi }(x)=\sup\left\{\phi (2^{j}x,0)/{\left|16\right|}^{j}:j\in \mathbb{N}\cup \{0\}\right\}$.

**Proof**

Putting *y* = 0 in (65), we get

(67) $$\begin{array}{c} ||{\left(m-1\right)}^{2}f\left(2x\right)-16{\left(m-1\right)}^{2}f\left(x\right)||\leq \phi \left(x,0\right) \end{array}$$

for all *x* ∈ *𝒳*. Thus we have

(68) $$\begin{array}{c} ||f\left(2x\right)-16f\left(x\right)||\leq \frac{1}{{\left|m-1\right|}^{2}}\phi \left(x,0\right) \end{array}$$

for all *x* ∈ *𝒳*. Replacing *x* by 2^*n*^ *x* in (68) and then dividing both sides by |16|^*n*+1^, we have

(69) $$\begin{array}{c} ||\frac{1}{16^{n+1}}f\left(2^{n+1}x\right)-\frac{1}{16^{n}}f\left(2^{n}x\right)||\leq \frac{1}{{\left|m-1\right|}^{2}{\left|16\right|}^{n+1}}\phi \left(2^{n}x,0\right) \end{array}$$

for all *x* ∈ *𝒳* and all nonnegative integers *n*. Thus the sequence {*f*(2^*n*^ *x*)/16^*n*^} is Cauchy by (64) and (69). Due to the completeness of *𝒴* as a non-Archimedean space, there exists a mapping *Q* so that

(70) $$\begin{array}{c} \underset{n\to \infty }{\lim}\frac{f\left(2^{n}x\right)}{16^{n}}=Q\left(x\right). \end{array}$$

For each *x* ∈ *𝒳* and non-negative integers *n*, we have

(71) ||f(x)−f(2nx)16n|| =||∑j=0n−1(f(2jx)16j−f(2j+1x)16j+1)|| ≤max⁡{||f(2jx)16j−f(2j+1x)16j+1||:0≤j<n} ≤1|16(m−1)2|max⁡{ϕ(2jx,0)|16|j:0≤j<n}.

Taking *n* → *∞* in (71) and applying (70), we can see that the inequality (66) holds when *m* ≥ 2. It follows from (64), (65), and (70) that, for all *x*, *y* ∈ *𝒳*,

(72) ||𝒟mQ(x,y)||=lim⁡n→∞1|16|n||𝒟mf(2nx,2ny)||≤lim⁡n→∞1|16|nϕ(2nx,2ny)=0.

Hence, the mapping *Q* satisfies (5). Now, let *Q*′ : *𝒳* → *𝒴* be another quartic mapping satisfying (66). Then we have

(73) ||Q(x)−Q′(x)|| =lim⁡k→∞1|16|k||Q(2kx)−Q′(2kx)|| ≤lim⁡k→∞1|16|kmax⁡{||Q(2kx)−f(2kx)||,||f(2kx)−Q′(2kx)||} ≤1|16(m−1)2|lim⁡k→∞lim⁡   n→∞max⁡{ϕ(2jx,0)|16|j:k≤j<n+k} =1|16(m−1)2|lim⁡k→∞sup⁡{ϕ(2jx,0)|16|j:k≤j<∞}=0

for all *x* ∈ *𝒳*. This shows the uniqueness of *Q*.

**Corollary 13**

Let *α* > 0, *𝒳* be a non-Archimedean space and let Γ : [0, *∞*)→[0, *∞*) be a function satisfying Γ(|*r*|*s*) ≤ Γ(|*r*|)Γ(*s*) for all *r*, *s* ∈ [0, *∞*) for which Γ(|2|) < |16|. Suppose that *f* : *𝒳* → *𝒴* is a mapping satisfying the inequality

(74) $$\begin{array}{c} ||{𝒟}_{m}f\left(x,y\right)||\leq \alpha \left(\Gamma \left(||x||\right)+\Gamma \left(||y||\right)\right) \end{array}$$

for all *x*, *y* ∈ *𝒳*, where *m* is an integer with *m* ≥ 2. Then there exists a unique quartic mapping *Q* : *𝒳* → *𝒴* such that

(75) $$\begin{array}{c} ||f\left(x\right)-Q\left(x\right)||\leq \frac{\alpha \Gamma \left(||x||\right)}{\left|16{\left(m-1\right)}^{2}\right|} \end{array}$$

for all *x* ∈ *𝒳*.

**Proof**

Defining *ϕ* : *𝒳* × *𝒳* → [0, *∞*) by *ϕ*(*x*, *y*) = *α*(Γ(||*x*||) + Γ(||*y*||)), we have

(76) $$\begin{array}{c} \underset{n\to \infty }{\lim}\frac{1}{{\left|16\right|}^{n}}\phi \left(2^{n}x,2^{n}y\right)\leq \underset{n\to \infty }{\lim}{\left(\frac{\Gamma \left(\left|2\right|\right)}{\left|16\right|}\right)}^{n}\phi \left(x,y\right)=0 \end{array}$$

for all *x*, *y* ∈ *𝒳*. We also have

(77) ϕ~(x)=sup⁡{ϕ(2jx,0)|16|j:0≤j<∞}=ϕ(x,0)=α(Γ(||x||))

for all *x* ∈ *𝒳*. Now, Theorem 12 implies the desired result.

We have the following result which is analogous to Theorem 12 for the functional equation (5).

**Theorem 14**

Let *ϕ* : *𝒳* × *𝒳* → [0, *∞*) such that

(78) $$\begin{array}{c} \underset{k\to \infty }{\lim}{\left|16\right|}^{k}\phi \left(\frac{x}{2^{k}},\frac{y}{2^{k}}\right)=0 \end{array}$$

for all *x*, *y* ∈ *𝒳*. Suppose that *f* : *𝒳* → *𝒴* is a mapping satisfying the inequality

(79) $$\begin{array}{c} {||{𝒟}_{m}f\left(x,y\right)||}_{𝒴}\leq \phi \left(x,y\right) \end{array}$$

for all *x*, *y* ∈ *𝒳*, where *m* is an integer with *m* ≥ 2. Then there exists a unique quartic mapping *Q* : *𝒳* → *𝒴* such that

(80) $$\begin{array}{c} ||f\left(x\right)-𝒬\left(x\right)||\leq \frac{1}{{\left|m-1\right|}^{2}}\tilde{\phi }\left(x\right) \end{array}$$

for all *x* ∈ *𝒳* where $\tilde{\phi }(x)=\sup\left\{{\left|16\right|}^{j}\phi (x/2^{j+1},0):j\in \mathbb{N}\cup \{0\}\right\}$.

**Proof**

In a similar way to the proof of Theorem 12, we have

(81) $$\begin{array}{c} ||f\left(2x\right)-16f\left(x\right)||\leq \frac{1}{{\left|m-1\right|}^{2}}\phi \left(x,0\right) \end{array}$$

for all *x* ∈ *𝒳*. If we replace *x* by *x*/2^*n*+1^ in the above inequality and multiply both sides of (81) to  |16|^*n*^, we get

(82) $$\begin{array}{c} ||16^{n}f\left(\frac{x}{2^{n}}\right)-16^{n+1}f\left(\frac{x}{2^{n+1}}\right)||\leq \frac{{\left|16\right|}^{n}}{{\left|m-1\right|}^{2}}\phi \left(\frac{x}{2^{n+1}},0\right) \end{array}$$

for all *x* ∈ *𝒳* and all non-negative integers *n*. Thus, we conclude from (78) and (82) that the sequence {2^*n*^ *f*(*x*/2^*n*^)} is Cauchy. Since the non-Archimedean space *𝒴* is complete, this sequence converges in *𝒴* to the mapping *Q*. Indeed,

(83) $$\begin{array}{c} Q\left(x\right)=\underset{n\to \infty }{\lim}16^{n}f\left(\frac{x}{2^{n}}\right),\;\left(x\in 𝒳\right). \end{array}$$

Using induction and (81), one can show that

(84) ||f(x)−16nf(x2n)|| ≤1|m−1|2max⁡{|16|jϕ(x2j+1,0):0≤j<n}

for all *x* ∈ *𝒳* and non-negative integers *n*. Since the right hand side of inequality (84) goes to 0 as *n* → *∞*, by applying (83), we deduce inequality (80). Now, in a similar way to the proof of Theorem 12, we can complete the rest of the proof.

**Corollary 15**

Let *α* > 0, *𝒳* be a non-Archimedean space and let Γ : [0, *∞*)→[0, *∞*) be a function satisfying Γ(|*r*|*s*) ≤ Γ(|*r*|)Γ(*s*) for all *r*, *s* ∈ [0, *∞*) for which Γ(|2|^−1^) < |16|^−1^. Suppose that *f* : *𝒳* → *𝒴* is a mapping satisfying the inequality

(85) $$\begin{array}{c} ||{𝒟}_{m}f\left(x,y\right)||\leq \alpha \left(\Gamma \left(||x||\right)+\Gamma \left(||y||\right)\right) \end{array}$$

for all *x*, *y* ∈ *𝒳*, where *m* is an integer with *m* ≥ 2. Then there exists a unique quartic mapping *Q* : *𝒳* → *𝒴* such that

(86) $$\begin{array}{c} ||f\left(x\right)-Q\left(x\right)||\leq \frac{\alpha \Gamma \left(||x/2||\right)}{{\left|m-1\right|}^{2}} \end{array}$$

for all *x* ∈ *𝒳*.

**Proof**

The proof is a direct consequence of Theorem 14 and similar to the proof of Corollary 13.
